# AGAPE (Automated Genome Analysis PipelinE) for Pan-Genome Analysis of *Saccharomyces cerevisiae*

**DOI:** 10.1371/journal.pone.0120671

**Published:** 2015-03-17

**Authors:** Giltae Song, Benjamin J. A. Dickins, Janos Demeter, Stacia Engel, Barbara Dunn, J. Michael Cherry

**Affiliations:** 1 Department of Genetics, Stanford University School of Medicine, Stanford, California, United States of America; 2 School of Science and Technology, Nottingham Trent University, Nottingham, United Kingdom; University of Strasbourg, FRANCE

## Abstract

The characterization and public release of genome sequences from thousands of organisms is expanding the scope for genetic variation studies. However, understanding the phenotypic consequences of genetic variation remains a challenge in eukaryotes due to the complexity of the genotype-phenotype map. One approach to this is the intensive study of model systems for which diverse sources of information can be accumulated and integrated. *Saccharomyces cerevisiae* is an extensively studied model organism, with well-known protein functions and thoroughly curated phenotype data. To develop and expand the available resources linking genomic variation with function in yeast, we aim to model the pan-genome of *S*. *cerevisiae*. To initiate the yeast pan-genome, we newly sequenced or re-sequenced the genomes of 25 strains that are commonly used in the yeast research community using advanced sequencing technology at high quality. We also developed a pipeline for automated pan-genome analysis, which integrates the steps of assembly, annotation, and variation calling. To assign strain-specific functional annotations, we identified genes that were not present in the reference genome. We classified these according to their presence or absence across strains and characterized each group of genes with known functional and phenotypic features. The functional roles of novel genes not found in the reference genome and associated with strains or groups of strains appear to be consistent with anticipated adaptations in specific lineages. As more *S*. *cerevisiae* strain genomes are released, our analysis can be used to collate genome data and relate it to lineage-specific patterns of genome evolution. Our new tool set will enhance our understanding of genomic and functional evolution in *S*. *cerevisiae*, and will be available to the yeast genetics and molecular biology community.

## Introduction

The first completed eukaryotic genome sequence was that of the budding yeast *Saccharomyces cerevisiae* strain S288C, completed through the effort of a worldwide sequencing consortium [1]. Since that time, many *S*. *cerevisiae* genomes have been sequenced, encompassing a wide variety of commercial and laboratory strains, as well as wild isolates. With next-generation sequencing methods becoming ubiquitous, whole genomes are now being analyzed *en masse*. This has led to interesting work on the relationship between genotype and phenotype. For example, in the studies of the adaptive evolution of freezing tolerance, Fay *et al*. [2] determined that an isolate taken from the soil beneath an oak tree in a natural woodland area in southern Pennsylvania (YPS163) is freeze tolerant, a phenotype associated with its increased expression of aquaporin AQY2 [3, 4]. Similarly, Doniger *et al*. [3] studied an Italian vineyard isolate (M22), and confirmed the presence of a reciprocal translocation between chromosomes VIII and XVI (relative to the laboratory strain S288C); this translocation is common in wine strains, and results in increased sulfite resistance, an adaptive trait for the yeast since vineyards are routinely dusted with elemental sulfur as a fungicide [5]. Argueso *et al*. [6] determined that a widely used Brazilian bioethanol strain that is resistant to heat and oxidative stress contains well-characterized alleles at several genes known to be linked with thermotolerance and fermentation performance. Novo *et al*. [7] studied a well-known commercial winemaking strain (EC1118) and found three unique regions on three different chromosomes containing 34 genes related to key fermentation characteristics, such as metabolism and transport of sugar or nitrogen. They also noted that >100 genes in the reference strain S288C are absent from the EC1118 genome. Comparative genomics work has revealed patterns of genetic variation including single nucleotide polymorphisms, and large-scale insertions and deletions in several wine and ale strain genomes [8]. Functional genomic analysis has also been undertaken in a saké yeast strain (K7), which has two large inversions and dozens of novel open reading frames (ORFs) compared to reference strain S288C [9].

Genomic variation in *S*. *cerevisiae* genomes, such as single-nucleotide polymorphisms (SNPs), small insertions/deletions (indels), and structural variation, have been investigated [10]. Despite much effort, the association of genomic variations with phenotype and functional annotations remains challenging, partly due to difficulties gaining accurate phenotypic information and obtaining genome sequences at high quality. Fortunately, because of its status as both a model organism and as an important industrial organism, many different *S*. *cerevisiae* strains have been intensively studied at the phenotypic, genetic and genomic levels and the resulting information has been extensively curated in the *Saccharomyces* Genome Database (SGD) [11, 12] (See S4 Table). Genomics studies using the standard S288C yeast reference genome have produced many informative and interesting results [13]. However, our understanding of yeast genetics and systems biology will widen and deepen if we can integrate new data into a pan-genome model to account for a greater proportion of the genetic and phenotypic variation exhibited by the global population of *S*. *cerevisiae*. A pan-genome is defined as the set of all genes in a species [14], and can be constructed from the union of gene sets over all *S*. *cerevisiae* strains.

The development and rapid expansion in the use of Next-Generation Sequencing (NGS) technologies has created an increase in the volume of high-throughput data. The expanding use of targeted approaches such as DNA-seq, RNA-seq, and ChIP-seq has also increased the types of data available. These developments allow questions and assumptions in population genetics and evolutionary biology to be addressed directly, but fulfilling the potential of these approaches depends on accurate and reproducible data analysis. Many computational methods are designed to handle DNA-seq data for assembly, annotation, and variation detection. However setting up a pipeline for these computational analyses is a non-trivial task. Existing analysis software often produces incongruent results even when addressing the same problems with the same data. Pipelines for the pan-genome analysis of bacteria have been developed such as PGAP [15], but these are not suitable for eukaryotic genomes, even for unicellular eukaryotes such as yeasts, which exhibit more complex gene structures and non-genic regions than prokaryotes. The frenetic pace at which new genomes are being sequenced has laid the groundwork for great steps forward in our understanding of chromosomal evolution and the extreme variability of the eukaryotic genome. However, the sheer volume of data presents a clear challenge because it has been, and is being, produced by different research groups using different techniques for sequence assembly, feature annotation, and gene functional analysis. Before we can realize the full potential of these new data and derive maximal benefit from the ever-increasing number of sequenced genomes generated by disparate groups, we must address the pressing need for a common standardized approach to genomic data analysis. To that end, we report here the development of AGAPE: an Automated Genome Analysis PipelinE for *S*. *cerevisiae*. The pipeline includes assembly, annotation, and variation-calling steps for the genome sequence of a given strain and generates integrative analyses among strains. We have sequenced, or re-sequenced, and analyzed the genomes of 25 *S*. *cerevisiae* strains that are commonly used in yeast laboratory research (S4 Table) to initiate analysis of the yeast pan-genome using AGAPE.

Simple eukaryotes such as fungi evolve rapidly and show presence or absence of genes in different populations within a single species [16, 17]. Our initial work can accelerate the establishment of the yeast pan-genome using AGAPE as more genome sequences are released [17]; assembly and annotation data from new strains can be used to continuously update the pan-genome, and the integrative analysis steps of the pipeline can be easily performed using the updated pan-genome. AGAPE can also be useful for biologists with limited bioinformatics expertise who can conduct computational analyses with their eukaryotic genomic data. Replacement software for a specific computational step can be easily plugged into the pipeline. All analyses, data, and the software pipeline reported here are freely available online, see Table 1.

**Table 1:** Location for the data, the software pipeline, and resources required for setting up the pipeline.

	Location	URL
AGAPE pipeline	GitHub	http://github.com/yeastgenome/AGAPE
Sequence raw reads in FASTQ	NCBI GenBank (BioProject: PRJNA260311)	http://www.ncbi.nlm.nih.gov/bioproject/PRJNA260311
Sequence assemblies and annotation data	SGD download site—Sequence Strains section	http://www.yeastgenome.org/download-data/sequence
All processed results found in this paper	SGD download site—Published Datasets section	http://www.yeastgenome.org/download-data/published-datasets
*S*.*cerevisiae* protein dataset used for AGAPE annotation	SGD Download site	http://downloads.yeastgenome.org/sequence/S288C_reference/orf_protein/
Expressed sequencing tag (EST) data used for AGAPE annotation	FungiDB	http://fungidb.org/common/downloads/
Fungi protein dataset used for AGAPE annotation	Ensembl Fungi	http://fungi.ensembl.org/

## Materials and Methods

### Strain sequences and genome assemblies

Twenty-five strains were selected for analysis based in part on their frequent use in genetic research (Table 2, S4 Table). The libraries were sequenced using Illumina HiSeq 2000, resulting in paired-end reads of 101 nucleotides each.

**Table 2:** Short description and assembly statistics of the 25 *S*. *cerevisiae* strains explored in this study.

Name	Description	Fold coverage	Number of scaffolds	Assembly size	Longest scaffold	Scaffold N50	Ploidy	Refe-rence
YS9^+^	Singapore baking strain	100	1972	11750421	142656	30314	Diploid	[10]
YPS163^+^	Pennsylvania woodland isolate	96	959	11692983	170627	39876	Diploid	[2]
YPS128^+^	Pennsylvania woodland isolate	95	1067	11608384	143401	39695	Diploid	[10]
YJM339^+^	Clinical isolate	102	994	11683869	216801	47674	Diploid	[18]
Y55*	Laboratory strain	112	829	11700636	406493	107844	Diploid	[19]
DBVPG6044^+^	West African isolate	176	819	11642411	134064	36171	Diploid	[10]
SK1*	Laboratory strain	261	978	11687249	326823	103064	Diploid	[20]
BC187^+^	California wine barrel isolate	177	853	11539626	135217	36331	Diploid	[10]
K11^+^	Saké strain	189	692	11532471	244353	47234	Diploid	[21]
L1528^+^	Chilean wine strain	186	692	11640535	150051	42013	Diploid	[10]
RedStar*	Commercial baking strain	180	1812	12003693	319971	98298	Diploid	URL^a^
UWOPS05_217_3^+^	Environmental isolate	57	1508	11398116	57541	13931	Diploid	[10]
FY1679*	S288C-derivative laboratory strain	329	886	11701731	454382	122764	Diploid	[22]
YPH499*	S288C-congenic laboratory strain	69	749	11721435	462932	125709	Haploid	[23]
RM11–1A*	Haploid derivative of California vineyard isolate	197	615	11571262	540496	114595	Haploid	[24]
10560–6B*	Sigma1278b-derivative laboratory strain	191	875	11642710	458709	109268	Haploid	[25]
BY4742*	S288C-derivative laboratory strain	103	868	11674767	341843	108974	Haploid	[26]
BY4741*	S288C-derivative laboratory strain	209	864	11678362	454112	112644	Haploid	[27]
FL100*	Laboratory strain	184	942	11667748	580633	118714	Haploid	[28]
W303*	Laboratory strain	301	967	11704989	336272	102309	Haploid	[29]
CEN.PK2–1Ca*	Laboratory strain	89	850	11651483	334215	115163	Haploid	[30]
SEY6210*	Laboratory strain	106	805	11664136	389964	122714	Haploid	[31]
X2180–1A*	S288C-derivative laboratory strain	112	904	11693006	298290	105189	Haploid	[32]
D273–10B*	Laboratory strain	112	866	11708626	343062	108887	Haploid	[33]
JK9–3d*	Laboratory strain	154	933	11669230	320854	103867	Haploid	[34]

^+^ Libraries made using 500–600 bp random shearing of genomic DNA

* Libraries made using Nextera tagmentation of genomic DNA

^a^ http://lesaffre-yeast.com/

We sequenced the libraries to high coverage ranging from 60- to 330-fold. Reads with low quality or ambiguous bases were discarded using the error correction program SGA (command line ‘sga correct-k 41—discard—learn’ version 0.9.35) [35]. In this error correction step, on average 2–3% of the raw reads were removed. Note that the preprocessing step before running the assembler program is important for assembly quality control [36]. The filtered reads were assembled to contigs using the *de novo* assembler program ABySS (command line ‘abyss-pe aligner = map k = 41’, version 1.3.4) [37]. The resulting contigs were extended to scaffolds using an SGA scaffolding pipeline (command lines ‘sga-align; sga-bam2de.pl-n 5-m 100-mina 95; sga-astat.py-m 100; sga scaffold-m 100—pe; sga scaffold2fasta-m 100—write-unplaced—use-overlap’) [35]. If desired, alternative parameters can be specified for each program in this assembly process.

### Gene annotations

Predictions of protein-coding genes (ORFs) were made using a combination of two methods: a homology-based approach and *ab initio* prediction. For the first approach we used the Chain and Net program [38] to find all intervals in each strain that are homologous to the reference genome. Next, for each matching interval we ran a modified version of the annotation utility program included in CHAP2 (The Cluster History Analysis Package). CHAP2 [39] uses LASTZ [40] for aligning the matching regions to the reference ORF sequences. We used the thoroughly curated SGD reference annotations, and predicted gene structures of each homologous ORF using AUGUSTUS (http://augustus.gobics.de). We replaced a component of CHAP2 (http://www.bx.psu.edu/miller_lab/dist/CHAP/README), the Wise2 (http://www.ebi.ac.uk) program with AUGUSTUS, because Wise2 is no longer available.

AGAPE also includes an *ab initio* annotation pipeline, called MAKER [41]. Protein and expressed sequence tag (EST) data for *S*. *cerevisiae*, required for running MAKER, were downloaded from SGD (http://www.yeastgenome.org) and FungiDB (http://fungidb.org) respectively (Table 1).

Results from the CHAP2 and MAKER methods were combined as follows: ORFs predicted by either method were kept. Predicted ORFs that lacked start or stop codons were discarded. Overlapping ORFs with the same stop coordinates but with potential alternative start sites were treated as separate annotations. ORFs predicted to have multiple exons were verified to include either the highly conserved splicing branch point 5’-UACUAAC-3’ or any of the unusual branch points CACUAAC, GACUAAC, UGCUAAC, AACUAAC, UAUUAAC, and AAUUAAC [42]. If no branch point consensus sequence could be identified within an intron, the ORF was discarded.

The nucleotide sequences of the predicted ORFs were compared against the S288C reference protein database using BLASTX [43]. Protein matches with e-values less than 1E-6, no more than 5% sequence length difference between the query and target ORFs, and sequence similarity greater than 90% were categorized as *bona fide* matches and were used to annotate the predicted ORFs. Predicted ORFs not matching these criteria were considered potential novel ORFs and were labeled ‘undefined’.

Regions within the contigs, which remained un-annotated or that were labeled ‘undefined’ in the initial phase of AGAPE were analyzed with the MAKER pipeline using all available fungal proteins (downloaded from http://fungi.ensembl.org) and ESTs (downloaded from http://fungidb.org). The resulting expanded dataset allowed us to capture more potential ORFs which were labeled with corresponding gene names. We applied the same procedure described above for predicted ORFs with potential alternative starts and for examining splicing branch point consensus sites for predicted ORFs with multiple exons. The remaining predicted ORFs were subjected to BLASTX analysis as above, but this time against all fungal proteins and ESTs, and the cutoff stringency was reduced (similarity > 80%).

Predicted ORFs that remained ‘undefined’ were consolidated with overlapping ORFs, and only ORFs greater than 300 bp were retained. All annotations are available in GFF3 format (http://www.sequenceontology.org/gff3.shtml) and the BLASTX output is available as a text file for each strain (http://www.yeastgenome.org/download-data/published-datasets).

### Identifying novel sequences and ORFs

Sequence reads for each strain in FASTQ format were aligned to the *S*. *cerevisiae* reference genome using Burrows-Wheeler Aligner (BWA) (‘bwa aln-q 15-l 35-k 2-n 0.04-o 2-e 6-t 1’ and ‘bwa sampe’) [44]. Unmapped reads were extracted using SAMtools programs ‘samtools sort’, and ‘samtools view’ (with parameter settings of ‘-u-f 4-F 264’, ‘-u-f 8-F 260’, and ‘-u-f 12-F 256’). Unmapped reads were assembled using ABySS with the same parameters as set in the whole genome assembly. The resulting contigs were aligned to the reference genome to confirm that they were not present in the reference. Contigs shorter than 300 bp, which is the length cutoff for predicted ORFs, were discarded because short contigs are more likely to be derived from reads of low quality, composed of ambiguous bases, or represent spurious ORFs. We consider the remaining contigs as new sequences that are not in the reference. These additional sequences were then aligned to their own strain’s whole genome assembly using LASTZ (version 1.03.02, with parameters ‘T = 2 Y = 3400’) to find the corresponding genomic region of each additional sequence in the whole genome assembly. We created a set of non-reference ORFs from each strain by collecting ORFs annotated for these additional genomic regions in the whole genome assembly.

### Integrative analyses of non-reference ORFs

The set of protein sequences of all the non-reference ORFs was aligned to itself to identify potential homologs using BLASTP with cutoff values (e-value less than 1E-1, sequence identity greater than 75%, and sequence length similarity greater than 75%, note we tested different cutoff values to choose the most appropriate combination in S3 Table). We made a binary matrix based on the pattern of presence or absence of each homologue group in each strain and used the matrix to calculate distance among 18 of the strains. The matrix did not include all 25 strains because we found no non-reference ORFs in 7 strains that are very closely related to the reference strain. Then we constructed a dendrogram of the 18 strains using ‘dist.gene’ and ‘nj’ functions in the ape R library (http://ape-package.ird.fr).

We also predicted molecular function associated with the non-reference ORFs using sequence similarity (BLASTP against NCBI Non-Redundant (nr) database, http://www.ncbi.nlm.nih.gov) and conserved protein domains using InterProScan [45].

### Variation identification and genome diversity

To identify SNPs and indels relative to the reference genome, we used the HugeSeq pipeline that integrates multiple variant calling programs [46]. We used the Phylogenetic Tree Galaxy tool (within Galaxy’s genome diversity section) [47] to infer a phylogenetic relationship and population structure based on the SNP data obtained by HugeSeq. To run this Galaxy tool, the SNP data was reformatted to gd_snp format and used as input for generating the phylogenetic tree and population structure to estimate relationship of the strains. Note the Phylogenetic Tree Galaxy tool includes filtering steps for discarding SNPs of low quality or SNPs that are in low coverage regions and we used default settings for these options.

### Tree construction of non-reference MAL gene family

The maltose catabolic, metabolic and transport genes (MAL) that are not part of the reference annotations were extracted from our non-reference features, from Bergstrom *et al*.*’s* [17] dataset, and from the NCBI Non-Redundant (nr) protein database using BLASTP with queries of the *MAL23*, *MAL43*, *MAL63*, and *MAL64* protein sequences. We constructed a maximum-likelihood tree of the non-reference MAL gene family using Phylogeny.fr with default parameters [48].

## Results and Discussion

### Overview of AGAPE

**Fig 1:**
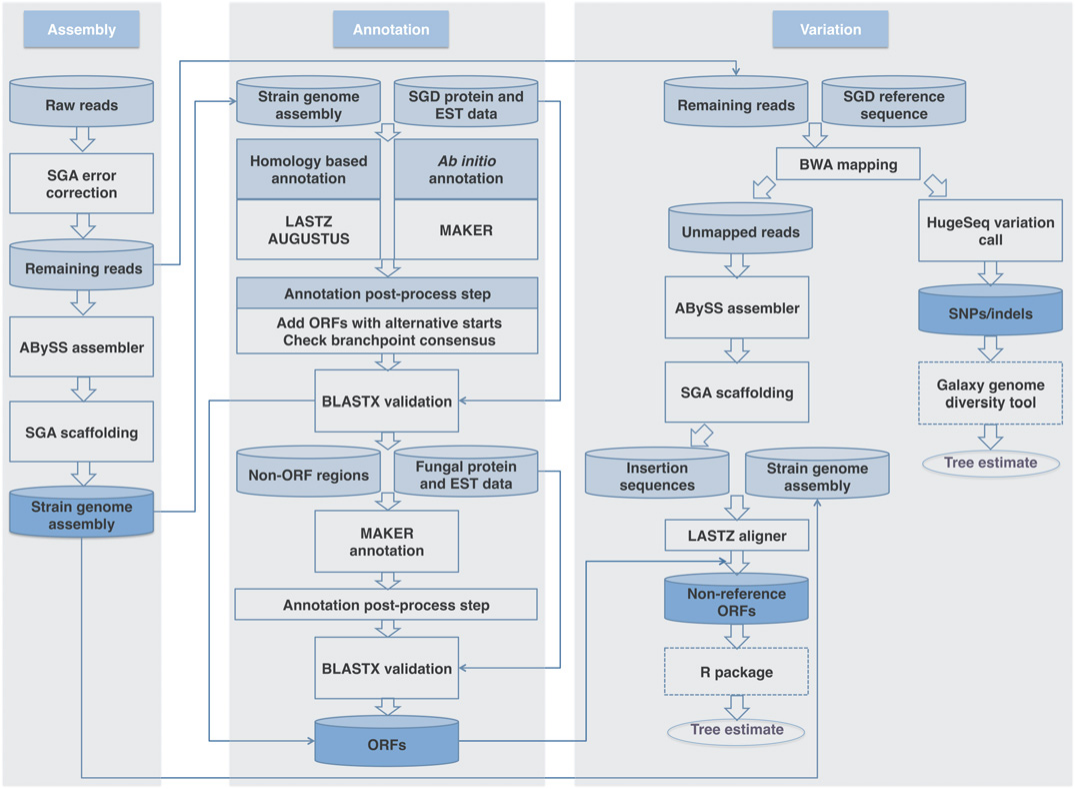
Pipeline overview of AGAPE for yeast. The pipeline consists of three parts; (a) assembly, (b) annotation, and (c) variation. Cylinder shapes indicate data, shaded cylinder final result data, arrows data flows, rectangular shapes programs, and dotted rectangular external package tools that are not included in our pipeline. After all ambiguous and low quality reads are discarded, the remaining reads are processed to generate assembly contigs (a). The assembly contigs from (a) are used as the input to annotate their genomic features including both reference ORFs inferred by a homology-based method and non-reference ORFs predicted by *ab initio* methods (b). Fungal (including yeast) protein and EST databases are used to accurately predict annotations. In a post-process annotation step, annotated ORFs are refined and corrected as shown in (b). For variation detections, the reads remaining after the error-correction step are mapped to the reference genome in (c). The procedure (c) then forks into two branches; one for unmapped and another for mapped reads. The unmapped reads are assembled in the manner described in (a) to contigs, then compared with the assembly contigs from (a) and annotation results from (b) to identify newly inserted sequences and ORFs that are not present in the reference genome. For the mapped reads, the mapping information is used for the HugeSeq pipeline that detects variations including SNPs relative to the reference. The SNP calls and the non-reference features identified in (c) can be used for further variation analysis using external tools, e.g. the Galaxy genome diversity tool and various R packages.

We created an integrated pipeline to discover the full set of genomic features of the *S*. *cerevisiae* species—the pan-genome—from whole-genome sequences of multiple strains. AGAPE consists of three main parts: assembly, annotation, and variation calls. Given the raw sequence reads of a given genome, a reference genome sequence, and reference genome annotations, the pipeline generates *de novo* assembly scaffolds and contigs, ORF annotations including non-reference ORFs, and sequence variation calls such as additional newly inserted sequences in the genome (not present in the reference genome) as well as SNPs relative to the reference. The whole pipeline is performed automatically as shown in Fig. 1 (for a detailed breakdown see the Materials and Methods section).

AGAPE was designed to generate genome assembly, annotation, and variation data, with features extracted from newly analyzed genomes added cumulatively to previously generated data. Integrative analyses can be done easily with the updated data and features. Although some organisms may not have thoroughly annotated reference genomes available, AGAPE can still generate the assembly and annotation data as long as a protein database is provided for predicting gene structure. (Note: although the NCBI Non-Redundant (nr) protein database can be attached to the AGAPE workflow, the speed of this annotation step is related to the number of sequences; we therefore recommend selecting a smaller protein database that includes only those proteins that are expected to be similar to the organism of interest). For the variation-calling steps, users can treat a subset of their contig-level sequences as the reference genome. The components of the pipeline can be easily substituted with alternative software as long as the input and output formats are similar to those used in the original step.

### Running the pipeline with the reference assembly for validation

**Fig 2:**
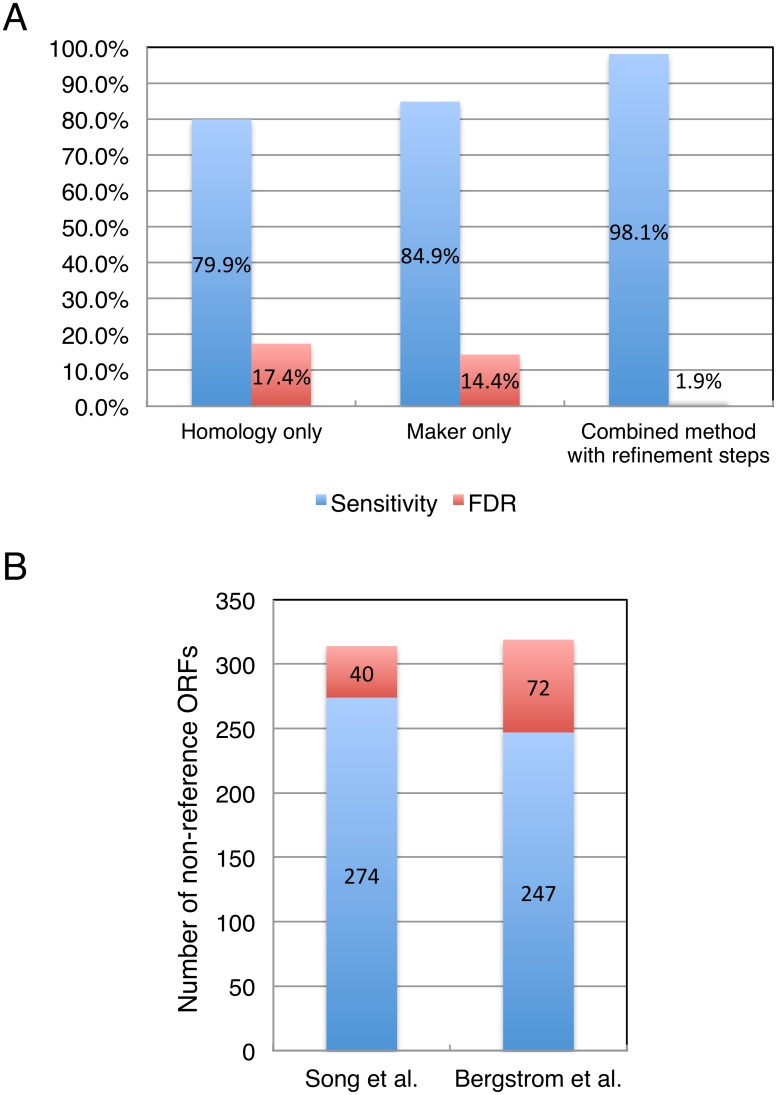
Pipeline validation based on annotation results. (A) Annotation accuracy of the pipeline is measured using the reference genome assembly as input. Whereas 80% of ORFs predicted by homology only are correct and 85% by MAKER only, our combined method with refinement steps predicts 98% of ORFs correctly. In terms of FDR, the combined method also shows better performance than the homology only or the MAKER only methods alone. (B) Annotation comparison of our non-reference ORFs to Bergstrom *et al*. [17] shows that 77% of 319 non-reference ORFs from Bergstrom *et al*. are commonly found in our results from 18 non-S288C strains. We identify 40 non-reference ORFs that were not identified by Bergstrom *et al*. [17] while Bergstrom *et al*. identify 72 non-reference ORFs not found in our study; these are presumably due to the non-overlapping strains among the sets of strains used in the two studies.

We validated the annotation steps by running the pipeline with the reference assembly as input (rather than FASTQ reads). These data were chosen because the reference assembly annotations have been thoroughly curated and can therefore be used to evaluate the accuracy of our predictions. We excluded true reference ORFs shorter than 300 bp to simplify the analysis (see Materials and Methods). Since annotation steps are designed to predict at most one ORF per locus, we also excluded some overlapping ORFs. When two ORFs have overlapping intervals and either one is classified as Dubious [49], the Dubious ORF was excluded. If both overlapping ORFs are Dubious, the shorter one was ignored. However overlapping ORFs are kept if both are classified as Verified ORFs. In total, we used 5684 reference ORFs as the “true” set. The annotation pipeline predicted 5638 ORFs, 5532 of which were identical to the reference annotations (98.1%). The FDR (False Discovery Rate) was therefore 1.88% ((5638–5532) / 5638). Our approach outperformed the use of either MAKER alone or the homology-based method alone (Fig. 2A), indicating that our pipeline can generate accurate annotation results if assemblies are of high quality.

### Genome sequences of 25 *S*. *cerevisiae* strains

To expand the *S*. *cerevisiae* pan-genome model, including those ORFs not present in the reference strain S288C, we sequenced strains that are commonly used in experimental yeast studies, including laboratory, wine, environmental, and clinical strains. (The strains are identified in Table 2 and S4 Table and short descriptions may be found at http://wiki.yeastgenome.org/index.php/Commonly_used_strains). Note that some of the strains in our list overlap with strains analyzed in genotype- [10, 17] and phenotype-based studies [50]. Some strains are diploid (Table 2). Diploidy may not influence the identification of new features in the pan-genome, but other types of variation analysis may be affected by heterozygosity. We subjected the strain genomes to deep sequencing with coverage ranging from 60X to 320X. Although our assembly contigs are still fragmented with gaps in some genomic regions composed of repeat elements such as rDNA and subtelomeres (S2 Fig.), this high coverage improved the resulting assembly compared to previous yeast sequencing projects [10]. The assemblies yielded N50 values ranging from 30 kb to 125 kb (Table 2) with the longest scaffold reaching 580 kb.

### *S*. *cerevisiae* non-reference ORFs and their functional predictions

**Fig 3:**
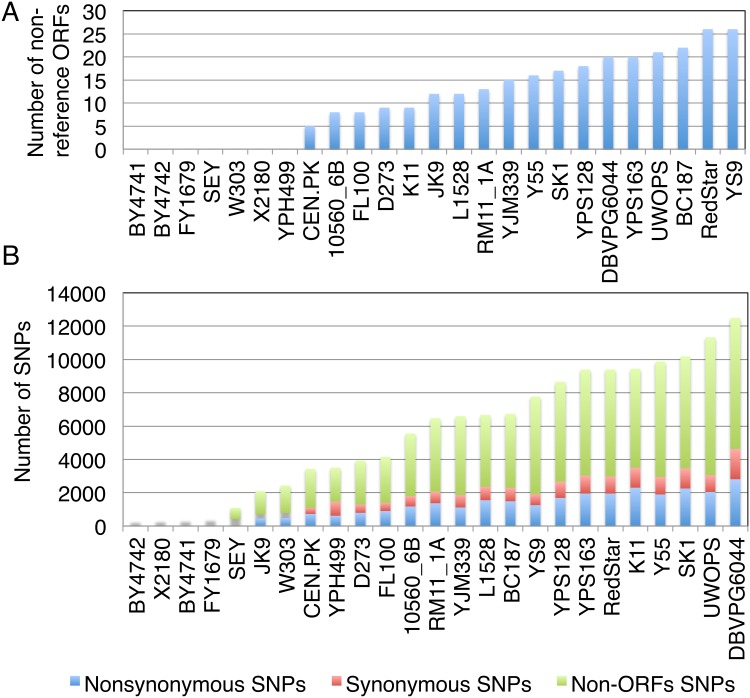
Variations in *S*. *cerevisiae* strains. (A) Number of non-reference ORFs in 25 *S*. *cerevisiae* strains. (B) Number of SNPs relative to the reference. According the number of SNPs, BY4742, X2180, BY4741, and FY1679 are essentially identical to the reference strain (S288C) and there are no non-reference ORFs in these strains. This supports the notion that these four strains are the same as S288C within experimental error. The variation patterns between non-reference ORFs and the number of SNPs show that strains that have more SNPs tend to have more non-reference ORFs, but there are some strains that have different patterns (e.g. K11 and YS9).

**Fig 4:**
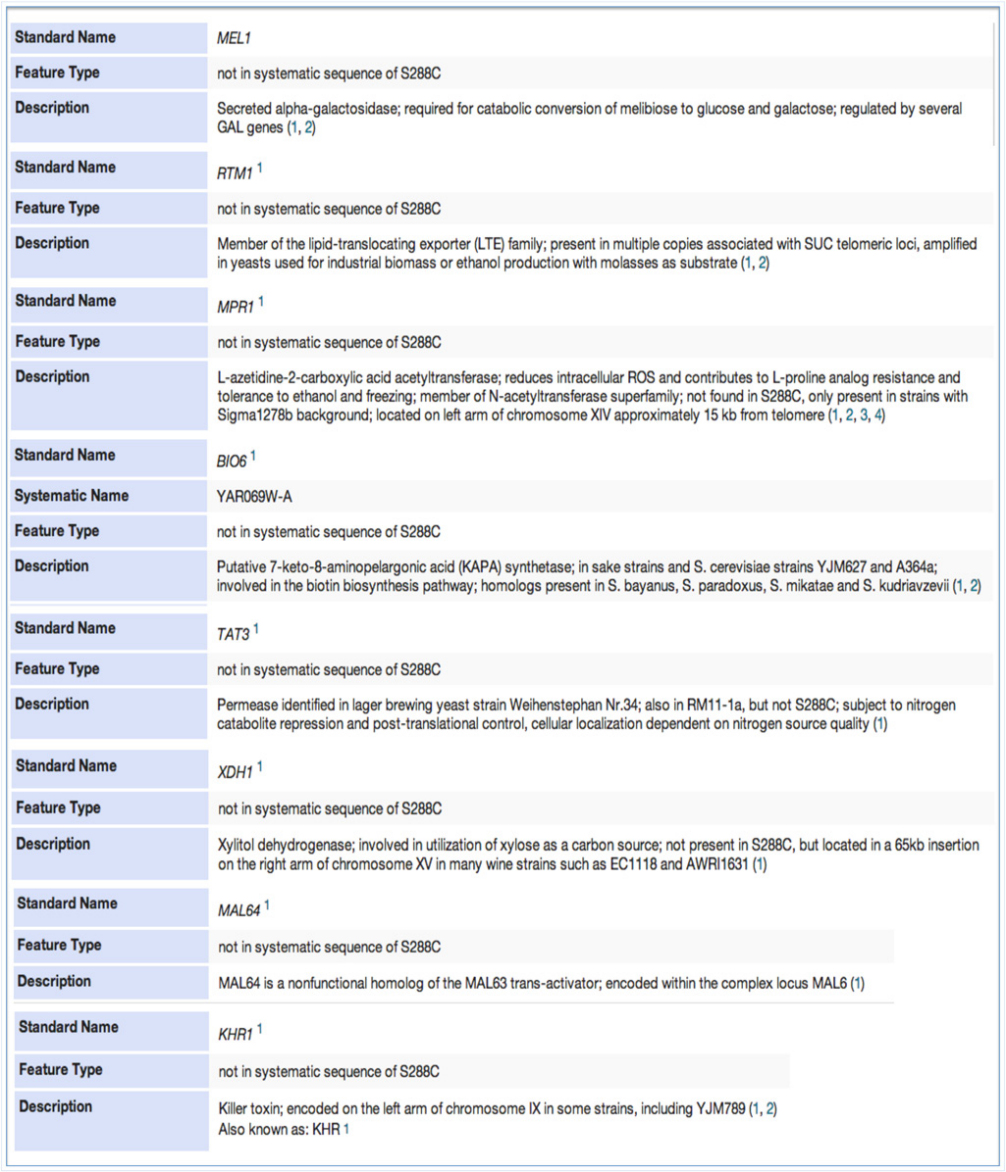
Known features not present in the reference genome. Annotations for 8 non-reference ORFs that were identified by our pipeline in 25 strains have been maintained in SGD. (a) *MEL1* in D273, FL100, JK9, and UWOPS. (b) *RTM1* in D273 and FL100. (c) *MPR1* in JK9, RedStar, and Y55. (d) *BIO6* in K11: K11 is a saké strain and this is consistent with the description that BIO6 is present in saké strains. (e) *TAT3* in RM11_1A, SK1, UWOPS, YPS128, and YPS163. (f) *XDH1* in RedStar and YS9. (g) *MAL64* in K11, UWOPS, YPS163, YPS128, and 10560–6B. (h) *KHR1* in BC187, YS9, FL100, YJM339, Y55, K11, YPS163, DBVPG6044, YPS128, and L1528.

As expected, we did not observe any non-reference ORFs among the seven strains (BY4741, BY4742, FY1679, SEY6210, JK9, W303, and X2180) known to be closely related to the S288C reference genome (Fig. 3A). Among the remaining 18 non-S288C strains, however, we found a total of 314 non-reference ORFs (Fig. 3A, S1 Table). We grouped the non-reference ORFs by aligning their protein sequences to each other using BLASTP. As a result, we identified 80 homologue groups of non-reference ORFs, including 16 unique ORFs that appear only in single strains (S1 Table). Eight ORFs out of the 80 non-reference groups were already annotated as non-reference features in SGD: *MEL1*, *RTM1*, *MPR1*, *BIO6*, *TAT3*, *XDH1*, *MAL64*, and *KHR1* (Fig. 4). Previous studies had shown the presence of the *BIO6* gene in saké strains and the *TAT3* gene in RM11; our AGAPE results recapitulate these results, showing *BIO6* occurring in the saké strain K11, and *TAT3* in RM11.

To predict functional association of the 80 non-reference ORF groups, we searched the NCBI Non-Redundant (nr) protein database using BLASTP and used InterProScan with the predicted protein sequences [28; S2 Table]. Comparing our set of non-reference ORFs to those found by similar studies, such as Bergstrom *et al*. [17], was instructive in showing how much of the pan-genome, as indicated by non-reference ORFs, our investigation has uncovered using AGAPE. Since annotation accuracy can be influenced by the quality of the *de novo* assembly, the comparison can also indirectly serve as an evaluation for the assemblies. Note that in the Bergstrom *et al*. [17] study, additional data such as low coverage paired-end Sanger sequences and genetic linkage were used to improve assembly while our pipeline used only *de novo* assembly. Of the 319 non-reference ORFs from Bergstrom *et al*. [17], 77% are shared with the non-reference ORFs identified by our pipeline (Fig. 2A). Forty non-reference ORFs from our 18 “non-S288C” strain genomes are not present in the Bergstrom *et al*. [17] analysis, while 72 ORFs from the Bergstrom *et al*. [17] study (coming from 14 strains that were mostly natural isolates and not represented in this study) were not found by our analysis. This supports the reasonable expectation that further sequencing will extend the pan-genome, especially if natural isolates are sequenced.

### SNP variations in the *S*. *cerevisiae* strains

SNPs identified relative to the reference genome for our 25 strains are shown in Fig. 3B. Strains BY4741, BY4742, FY1679, and X2180 all have less than 5 SNPs per 100,000 bp, indicating that they are essentially identical to S288C (the SGD reference genome). This is particularly important as FY1679 contributed roughly 50% of the initial chromosomal sequence released in 1996 [1]. Strains BY4741 and BY4742 are S288C-derivative strains were constructed to make an ORF deletion collection [51]. The variation between these strains and S288C was known to be miniscule (T. Yamaguchi and F. Roth, personal communication), and our results confirm this. These SNP-based results are also consistent with the fact that we did not find any non-reference ORFs in these four strains (see above section). In general, strains that have more non-reference ORFs also tend to contain more SNPs, especially in the laboratory strains (Fig. 3).

Interestingly, the two baking strains (YS9 and RedStar) have similar or lower numbers of SNPs relative to the S288C reference, compared to strains isolated from more natural environments (UWOPS, YPS163, YPS128, and DBVPG6044), indicating that the baking strains are less diverged from S288C than the natural environment strains (Fig. 3B). However, YS9 and RedStar contain the most non-reference features (26 ORFs among the 2 strains), *i*.*e*. they have more non-reference ORFs than any other environmental strains. A total of 15 non-reference ORFs are shared by both baking strains, and are not present in any other strains (Groups 51–64, S1 Table).

### Phylogenetic inferences and population structure of *S*. *cerevisiae*

**Fig 5:**
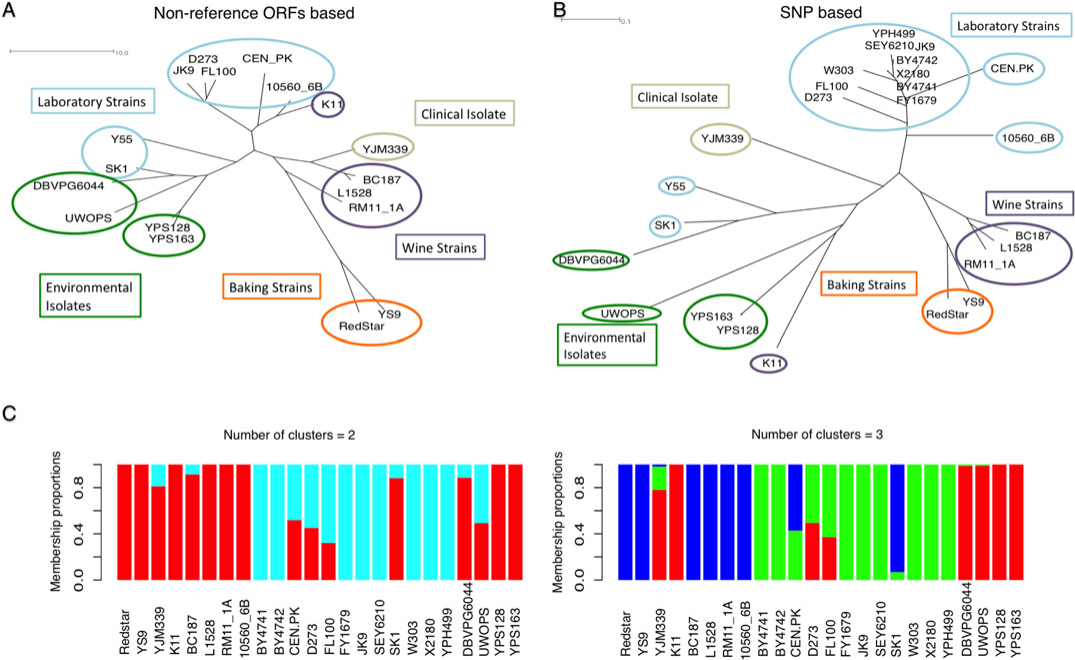
Phylogenetic inferences and population structure of *S*. *cerevisiae* strains from variation. (A) A neighbor-joining tree based on non-reference ORFs among 18 *S*. *cerevisiae* strains. (B) A neighbor-joining tree based on SNPs relative to the reference among 25 *S*. *cerevisiae* strains. The origin of each strain is indicated by the color of the enclosing circle. Strains that originated from similar sources appear close to each other in both trees, but there are some differences (e.g. SK1, K11, and YJM339). (C) Population structure based on SNPs using the Genome diversity tool in Galaxy. Statistical scores were also computed by the Galaxy tool in order to choose the most appropriate number of clusters (K). In our case, “K = 2 or 3” showed the lowest cross-validation error scores among the K values tested (with scores of 0.90 and 0.95, respectively). Colors were generated automatically and are not congruent with colors used in A and B.

A binary matrix based on patterns of presence or absence of the non-reference ORF groups in the 18 “non-S288C” strains that contained non-reference ORFs was used to calculate distance and construct a tree of the 18 strains based on a neighbor-joining method. This tree displays the relationships among the 18 strains based only on non-reference features (Fig. 5A). We also generated a tree based on the genome-wide SNPs found in each strain (relative to the reference). This tree reflects genomic distance based on the divergence of each strain from the reference, within only reference-homologous regions (Fig. 5B).

In both trees, strains isolated from similar environments are generally located closely together. For instance, the two baking strains (RedStar and YS9) are grouped together, as are the three vineyard/wine strains (RM11–1A, L1528, and BC187) and the two oak strains (YPS163 and YPS128). Lab strains that are close to S288C such as D273 and FL100 are grouped together in both trees close to the tightly-grouped S288C-related strains. Non-S288C-based laboratory strains, SK1 and Y55, used widely in studies of meiosis, appear as a branch off the lineage of environmental strain DBVP6044. Interestingly, K11 and YJM339 show different patterns in the two trees. A structure plot suggests the existence of mosaicism in several strains, with SK1 sharing cluster identity over most of its genome with baking and wine strains (Fig. 5C). This may be relevant to the unknown origin of laboratory strain SK1 and may indicate that SK1 has been mixed with other strains.

Unlike the vineyard/wine strains RM11–1A, L1528, and BC187, which are grouped together in both trees, the saké strain K11 appears close to laboratory strains CEN.PK and 10560–6B in the non-reference ORF-based tree, but in the SNP-based tree it clusters more closely to other environmental strains like YPS163 and YPS128, similar to results reported by Liti *et al*. [10]. Most non-reference ORFs of K11 are present in other environmental and wine strains.

### Case study of non-reference ORFs in strain K11

The distribution of 314 non-reference ORFs into 80 putatively homologue groups enables an exploratory analysis of ORFs that are absent from the reference strain.

As a means to link genotypes with phenotypes, strains used in the production of alcoholic beverages are of particular merit given the intense interest in understanding the metabolism of fermentation in these strains. Saké is made from a rice ferment known as koji; before a saké strain of *S*. *cerevisiae* can produce alcohols, the rice undergoes saccharification by a mold (or filamentous fungus, viz. *Aspergillus oryzae*) that metabolizes complex carbohydrates (starch) into sugars (glucose). Saké yeasts form a clade within *S*. *cerevisiae* [10, 52] and possess distinct features such as the ability to synthesize biotin [53].

In strain K11, a saké yeast, we have identified 10 non-reference ORFs belonging to 9 homologue groups (S1 Table). Consistent with biotin prototrophy in saké yeast strains, one of these (K11.ORF10) is identical at the DNA level (over its full length) with *BIO6* (GenBank AB188681.1). The *BIO6* gene is required for biotin biosynthesis and was identified in strain K7 from which K11 is derived [53].

**Fig 6:**
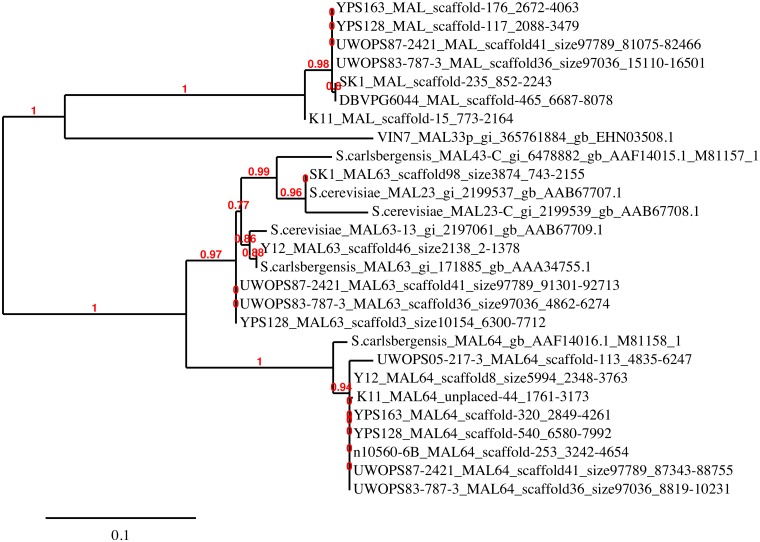
Phylogenetic tree of the non-reference *MAL* gene family. The *MAL23*, *MAL43*, *MAL63*, and *MAL64* genes are known non-reference features that may be associated with maltose activator function. We included all non-reference *MAL* activator genes identified in *S*. *cerevisiae* including sequences from this study, sequences from Bergstrom *et al*. [17], and ones deposited in the NCBI protein database. The *MAL* genes have been found in environmental and saké strains, but have not been detected in baking and European wine strains. One group of *MAL* genes in the upper part of the gene tree, detected in K11, YPS128, YPS163, UWOPS87, UWOPS83, SK1, and DBVPG6044 strains, is clustered separately from the other *MAL* genes.

At an intermediate stage of saké fermentation maltose is produced [54], potentially selecting for the retention, evolution, or horizontal acquisition of maltose utilization genes. Mutagenized strains of saké yeast with low maltose utilization appear to generate higher levels of malate [55], an organic acid contributing to the flavor of the beverage. Genes for maltose permease (GenBank BAB59002.1) and maltase (GenBank BAB59003.1) have been identified in *Aspergillus oryzae* and appear to be in a gene cluster with a regulatory gene [56]. Several maltose gene clusters are present in the *S*. *cerevisiae* pan-genome (Fig. 6). A maltose gene cluster such as *MAL6* typically consists of a maltose permease (*MAL61*), maltase (*MAL62*), and a *MAL* regulatory/activator gene (*MAL63*). Constitutively active forms of the regulatory proteins coded for by these genes have also been identified and appear to relate to loss of function mutations affecting C-terminal residues responsible for negative regulatory function [57]. At the *MAL6* locus an additional activator gene *MAL64* has been described [58]. A premature termination codon in *MAL64* confers constitutive expression [57] although the function of the wild-type allele is unclear.

One of the homologue groups identified by LASTZ is comprised of reading frames similar to *MAL* activator loci (see S2 Table). In saké strain K11, two ORFs fall into this group. K11.ORF1 shows partial similarity to maltose activator genes from multiple loci and its function therefore awaits further investigation, while K11.ORF9 shows substantial similarity (~98% at the DNA sequence level) to *MAL64*. An alignment (not shown) indicates that, across its length, K11.ORF9 closely resembles wild-type *MAL64* in other *S*. *cerevisiae*, and a phylogenetic tree (Fig. 6) indicates that the divergence between *MAL63* and *MAL64* regulatory genes preceded the divergence of multiple strains.

Another interesting non-reference homologue group is represented in K11 by K11.ORF8 and in SK1 by SK1.ORF11, both of which have 100% sequence identity with an epoxide hydrolase-like protein previously identified in saké strain K7 (GenBank GAA21449.1; [9, 59]). This ORF was previously identified in K7 and *S*. *paradoxus* and has a presumed bacterial origin [9], thus representing a possible trans-kingdom horizontal transfer; it has also been identified in 2 commercial wine strains, a sourdough strain and a fuel ethanol strain [59, 60]. Given the toxicity associated with reactive epoxide compounds and the presence of a seemingly non-homologous epoxide hydrolase in *Aspergillus oryzae* (GenBank XP_001727603.2), it is tempting to suppose that this ORF is required in the saké environment.

## Conclusion

Rapid evolution and the mosaic structure of genomes in microorganisms makes adequately capturing the diversity of a taxonomic group a difficult task, and requires systematic analysis of multiple genomes. Information from multiple bacterial isolates is frequently combined into a pan-genome, which comprises all genes found within a particular taxon. We have adopted this approach with yeast and have created a flexible pipeline, AGAPE, that uses a variety of tools and sources of information to construct and update a pan-genome. Although AGAPE generates assemblies that can be used to examine between-strain differences, a critical additional output is non-reference ORFs, and AGAPE identifies these by combining prediction methods.

We have explored the utility of this approach in yeast by using AGAPE to identify non-reference ORFs through analysis of high-throughput sequencing data from 25 *S*. *cerevisiae* genomes. This examination of a small set of non-reference ORFs within *S*. *cerevisiae* demonstrates that an updatable pan-genome model can be used as a starting point for analysis of function. We also found that contrasting patterns in SNP- and ORF-based phylogenies, combined with analysis of population structure, suggest that the dynamics of horizontal gene transfer, recombination, or gene gain and loss may be fruitfully investigated as more strains are sequenced and the pan-genome is expanded. Eventually it may be possible to characterize for a particular strain whether ORFs that are not part of the “core” genome (*i*.*e*., the set of genes shared by all *S*. *cerevisiae* strains) arose by retention and evolution (or duplication followed by divergent evolution) of ancestral genes, or by horizontal acquisition of “novel” genes, e.g., by mating with diverged *S*. *cerevisiae* strains or through interspecific hybridization.

Despite the difficulties in assembling complete chromosomes, which complicates determination of the presence or absence of some genomic loci, AGAPE provides an expandable pan-genome. The process includes thorough annotation and variation steps, and thus opens a new window to genotype-phenotype association studies. Analysis problems caused by the difficulty of generating complete assemblies, particularly in examination of repetitive elements, can be ameliorated by incorporating improved methods such as using mate-pair libraries and genetic linkage.

Beyond yeast, the AGAPE pipeline can be used for genome analyses of other eukaryotes. AGAPE can be modified to consider more complicated gene models and more sophisticated assembly methods can be used to investigate genomes rich in repetitive sequences. In addition, the steps defined in AGAPE can guide genomics studies for researchers who have little experience in computational biology. Our high-quality genome data and the analysis for 25 commonly studied strains are also important resources for furthering yeast genetics studies. The AGAPE package, genome annotation data, and the ongoing expansion of the yeast pan-genome model will facilitate genetic studies in this important model organism.

## Supporting Information

S1 Table 80 non-reference ORF groups. We classified 314 non-reference ORFs from the 18 non-S288C strain genomes into 80 homologue groups using BLASTX.
(PDF)

S2 Table Functional predictions for 80 non-reference ORF groups. Functional association of 80 novel ORFs were predicted using BLAST search and InterPro.
(PDF)

S3 Table Choosing appropriate cutoff values for constructing the phylogentetic tree based on presence or absence of novel genes. Different cutoff values were applied to construct the non-reference based trees in Fig. 5(A). The tree for each combination of the cutoff values was compared to the genome-wide SNP-based tree in Fig. 5(B) using Ktreedist. Lower K scores from Ktreedist indicate that two trees are more similar in terms of differences of the relative branch length and topology. BLAST E-value cutoff did not affect the tree topology. Similarity higher than 75% and length cutoff of 75% showed the lowest K score, so we chose 75% for both similarity and length cutoff values.
(PDF)

S4 Table Phenotype count per strain. We counted the number of phenotypes per strain as reported in SGD and chose to sequence those strains with the highest phenotype counts. Note that we have grouped all four of the S288C-identical strains (BY4741, BY4742, FY1679 and X2180) into one class called “S288C”. In addition to the strains listed in this table, we chose several other strains to sequence as described in the main text.
(PDF)

S1 Fig Copy number variants in S. cerevisiae. Copy number variants (CNVs) were called by the program “CNVnator” based on read coverage depth of each strain genome relative to the reference genome. Genomic intervals identified as CNVs in each strain were visualized as blue boxes using the IGV (Integrative Genomics Viewer) tool.
(PDF)

S2 Fig Assembly coverage in chromosome IV. All the assembly contigs of each strain genome were aligned to the reference genome using LASTZ. The alignments were visualized using the IGV tool. Since all the alignments for other chromosomes are available, users can easily view assembly coverage in other chromosomes with IGV.
(PDF)
