# Correction: AGAPE (Automated Genome Analysis PipelinE) for Pan-Genome Analysis of *Saccharomyces cerevisiae*

**DOI:** 10.1371/journal.pone.0129184

**Published:** 2015-05-27

**Authors:** Giltae Song, Benjamin J. A. Dickins, Janos Demeter, Stacia Engel, Jennifer Gallagher, Kisurb Choe, Barbara Dunn, Michael Snyder, J. Michael Cherry

Jennifer Gallagher, Kisurb Choe, and Michael Snyder are missing from the author list. Please view the correct author order, affiliations, and citation here:

Giltae Song ^1^, Benjamin J. A. Dickins ^2^, Janos Demeter ^1^, Stacia Engel ^1^, Jennifer Gallagher ^1^, Kisurb Choe ^1^, Barbara Dunn ^1^, Michael Snyder ^1^, J. Michael Cherry ^1^

1 Department of Genetics, Stanford University School of Medicine, Stanford, California, United States of America, 2 School of Science and Technology, Nottingham Trent University, Nottingham, United Kingdom

Song G, Dickins BJA, Demeter J, Engel S, Gallagher J, Choe K, et al. (2015) AGAPE (Automated Genome Analysis PipelinE) for Pan-Genome Analysis of *Saccharomyces cerevisiae*. PLoS ONE 10(3): e0120671. doi:10.1371/journal.pone.0120671

There are missing Author Contributions. The correct contributions are: Conceived and designed the experiments: GS MS JMC. Performed the experiments: GS JG KC BD. Analyzed the data: GS BJAD JD. Contributed reagents/materials/analysis tools: GS JG KC BD. Wrote the paper: GS BJAD JD SE BD JMC.

There is an omission in the Acknowledgments. The following sentence should be included in the Acknowledgments: We thank SGD Project staff for the creation of the high quality and detailed database of S. cerevisiae genes and their products and Webb Miller for helpful comments. Illumina sequencing services were performed by the Stanford Center for Genomics and Personalized Medicine.
