# Supplementary material for: AGAPE (Automated Genome Analysis PipelinE) for Pan-Genome Analysis of Saccharomyces cerevisiae
Source: PLoS One. 2015 Mar 17;10(3):e0120671. doi: 10.1371/journal.pone.0120671 (PMC4363492; doi:10.1371/journal.pone.0120671)
Supplement: S1 Table — We classified 314 non-reference ORFs from the 18 non-S288C strain genomes into 80 homologue groups using BLASTX. (PDF) [file pone.0120671.s001.pdf]

S1 Table. 80 non-reference ORF groups. We classified 314 non-reference ORFs from the 18 non-S288C strain genomes into 80 homologue groups using BLASTX.

Group number	ORF name
Group1	10560_6B.ORF1 10560_6B.ORF2 10560_6B.ORF3 10560_6B.ORF4 10560_6B.ORF6
Group2	10560_6B.ORF5 DBVPG6044.ORF11 K11.ORF1 K11.ORF9 SK1.ORF3 UWOPS.ORF1 YPS128.ORF1 YPS128.ORF14 YPS163.ORF8 YPS163.ORF9
Group3	10560_6B.ORF7 D273.ORF9 K11.ORF5 RedStar.ORF13
Group4	10560_6B.ORF8 CEN.PK.ORF5 DBVPG6044.ORF25 K11.ORF10
Group5	10560_6B.ORF9 YS9.ORF1
Group6	BC187.ORF1 BC187.ORF6 BC187.ORF14 L1528.ORF10 RM11_1A.ORF9 YJM339.ORF3 YS9.ORF6
Group7	BC187.ORF2 BC187.ORF7 BC187.ORF13 L1528.ORF9 RM11_1A.ORF10 YJM339.ORF4 YS9.ORF7
Group8	BC187.ORF3 BC187.ORF8 BC187.ORF12 L1528.ORF8 RM11_1A.ORF11 YJM339.ORF5
Group9	BC187.ORF4 L1528.ORF7 RM11_1A.ORF12 YJM339.ORF1 YS9.ORF4
Group10	BC187.ORF5 D273.ORF6 JK9.ORF1 L1528.ORF4 RedStar.ORF7 RM11_1A.ORF1 Y55.ORF17 YJM339.ORF13 YPS128.ORF4 YPS163.ORF1 YS9.ORF2
Group11	BC187.ORF9 DBVPG6044.ORF3 FL100.ORF9 K11.ORF3 L1528.ORF2 Y55.ORF8 YJM339.ORF6 YPS128.ORF8 YPS163.ORF3 YS9.ORF8
Group12	BC187.ORF10 L1528.ORF5 SK1.ORF2 Y55.ORF16 YJM339.ORF8
Group13	BC187.ORF11 DBVPG6044.ORF10 K11.ORF2 L1528.ORF6 RedStar.ORF32 RM11_1A.ORF13 Y55.ORF15 YJM339.ORF7 YS9.ORF3
Group14	BC187.ORF15 L1528.ORF11 RM11_1A.ORF8 YJM339.ORF2 YS9.ORF5
Group15	BC187.ORF16 YPS128.ORF7 YPS163.ORF12
Group16	BC187.ORF22 CEN.PK.ORF2 DBVPG6044.ORF2 DBVPG6044.ORF7 DBVPG6044.ORF26 L1528.ORF3 L1528.ORF12 UWOPS.ORF13 Y55.ORF14 YJM339.ORF14 YPS163.ORF21
Group17	BC187.ORF23 CEN.PK.ORF1 L1528.ORF13 UWOPS.ORF12 YJM339.ORF15 YPS128.ORF20 YPS163.ORF22
Group18	BC187.ORF24 L1528.ORF14
Group19	BC187.ORF25 L1528.ORF15
Group20	CEN.PK.ORF3 DBVPG6044.ORF22 SK1.ORF17
Group21	CEN.PK.ORF4 DBVPG6044.ORF23 SK1.ORF16

Group22	CEN.PK.ORF6 DBVPG6044.ORF1 RedStar.ORF9 SK1.ORF1 UWOPS.ORF10 Y55.ORF1 YPS128.ORF5
Group23	CEN.PK.ORF7 YJM339.ORF16
Group24	D273.ORF1 FL100.ORF1 JK9.ORF4 UWOPS.ORF9
Group25	D273.ORF2 FL100.ORF2 JK9.ORF5 UWOPS.ORF8
Group26	D273.ORF3 FL100.ORF3 JK9.ORF6 UWOPS.ORF7
Group27	D273.ORF4 FL100.ORF4 JK9.ORF7 UWOPS.ORF6
Group28	D273.ORF5 FL100.ORF5 JK9.ORF8 UWOPS.ORF5
Group29	D273.ORF7 DBVPG6044.ORF20 FL100.ORF7 SK1.ORF19 UWOPS.ORF22 Y55.ORF19
Group30	D273.ORF8 DBVPG6044.ORF21 FL100.ORF8 SK1.ORF18 UWOPS.ORF21 Y55.ORF18
Group31	DBVPG6044.ORF4 Y55.ORF11
Group32	DBVPG6044.ORF5 Y55.ORF12
Group33	DBVPG6044.ORF6 RM11_1A.ORF14 SK1.ORF15 UWOPS.ORF2 YPS128.ORF13 YPS163.ORF14
Group34	DBVPG6044.ORF8 SK1.ORF10 Y55.ORF6 YJM339.ORF9 YPS128.ORF12 YPS163.ORF10
Group35	DBVPG6044.ORF9 RedStar.ORF1 SK1.ORF5 UWOPS.ORF17 YPS128.ORF6 YPS163.ORF11
Group36	DBVPG6044.ORF12 Y55.ORF10
Group37	DBVPG6044.ORF13 SK1.ORF6 UWOPS.ORF3 Y55.ORF2 YJM339.ORF12 YPS128.ORF2 YPS128.ORF18 YPS163.ORF18
Group38	DBVPG6044.ORF14 DBVPG6044.ORF15 SK1.ORF7 SK1.ORF8 UWOPS.ORF16 Y55.ORF3 Y55.ORF4 YJM339.ORF11 YPS128.ORF3 YPS128.ORF17 YPS163.ORF19
Group39	DBVPG6044.ORF16 SK1.ORF9 UWOPS.ORF15 Y55.ORF5 YJM339.ORF10 YPS128.ORF16 YPS163.ORF20
Group40	DBVPG6044.ORF17 RedStar.ORF3 RedStar.ORF18 RM11_1A.ORF6 SK1.ORF12 UWOPS.ORF18 YPS128.ORF9 YPS163.ORF5 YS9.ORF24
Group41	DBVPG6044.ORF18 RedStar.ORF5 RM11_1A.ORF4 SK1.ORF13 UWOPS.ORF20 YPS128.ORF10 YPS163.ORF6
Group42	DBVPG6044.ORF19 RedStar.ORF6 RM11_1A.ORF3 SK1.ORF14 UWOPS.ORF14 YPS128.ORF11 YPS163.ORF7
Group43	DBVPG6044.ORF24 RM11_1A.ORF2 UWOPS.ORF23 Y55.ORF13 YJM339.ORF17 YJM339.ORF18
Group44	FL100.ORF6 JK9.ORF9
Group45	JK9.ORF3 YPS163.ORF2 Y55.ORF7
Group46	K11.ORF6 YPS128.ORF15 YPS163.ORF15
Group47	K11.ORF7 UWOPS.ORF4 YPS128.ORF19 YPS163.ORF16

Group48	K11.ORF8 SK1.ORF11
Group49	RedStar.ORF4 RM11_1A.ORF5 UWOPS.ORF19
Group50	RedStar.ORF10 RedStar.ORF12 YS9.ORF9 YS9.ORF12
Group51	RedStar.ORF11 YS9.ORF10
Group52	RedStar.ORF14 YS9.ORF28
Group53	RedStar.ORF15 YS9.ORF27
Group54	RedStar.ORF16 YS9.ORF26
Group55	RedStar.ORF17 YS9.ORF25
Group56	RedStar.ORF19 YS9.ORF23
Group57	RedStar.ORF20 YS9.ORF22
Group58	RedStar.ORF21 RedStar.ORF26 YS9.ORF17 YS9.ORF21
Group59	RedStar.ORF22 YS9.ORF20
Group60	RedStar.ORF24 YS9.ORF19
Group61	RedStar.ORF25 YS9.ORF18
Group62	RedStar.ORF27 YS9.ORF16
Group63	RedStar.ORF28 YS9.ORF15
Group64	RedStar.ORF30 YS9.ORF13
Group65	BC187.ORF17
Group66	BC187.ORF18
Group67	BC187.ORF19
Group68	BC187.ORF20
Group69	BC187.ORF21
Group70	K11.ORF4
Group71	RedStar.ORF2
Group72	RedStar.ORF23
Group73	RedStar.ORF29
Group74	RedStar.ORF31

Group75	RM11_1A.ORF15
Group76	SK1.ORF4
Group77	UWOPS.ORF11
Group78	YPS163.ORF4
Group79	YPS163.ORF13
Group80	YS9.ORF14
