# Supplementary material for: AGAPE (Automated Genome Analysis PipelinE) for Pan-Genome Analysis of Saccharomyces cerevisiae
Source: PLoS One. 2015 Mar 17;10(3):e0120671. doi: 10.1371/journal.pone.0120671 (PMC4363492; doi:10.1371/journal.pone.0120671)
Supplement: S2 Table — Functional association of 80 novel ORFs were predicted using BLAST search and InterPro. (PDF) [file pone.0120671.s002.pdf]

S2 Table. Functional predictions for 80 non-reference ORF groups. Functional association of 80 novel ORFs were predicted using BLAST search and InterPro.

	Top hit in BLAST search (NCBI GenBank and subject title)	Function (InterPro prediction)	Process (InterPro prediction)	Location (InterPro prediction)
Group1	WP_001231131 ATP phosphoribosyltransferase [Salmonella enterica]	ATP phosphoribosyltransferase activity|GO:0003879	histidine biosynthetic process|GO:0000105	
Group2	AAF14016 MAL activator protein [Saccharomyces carlsbergensis]	transcription factor activity|GO:0000981	regulation of transcription, DNA-templated|GO:0006355	nucleus|GO:0005634
Group3	EGA60513 hypothetical protein FOSTERSO_4311 [Saccharomyces cerevisiae FostersO			
Group4	BAD89998 Biotin biosynthesis enzyme [Saccharomyces cerevisiae]	adenosylmethionine-8-amino-7-oxononanoate transaminase activity|GO:0004015	biotin biosynthetic process|GO:0009102	
Group6	CAY82272;EGA73576;EGA73583;EGA77667;AEJ82630 EC1118_1N26_0023p [Saccharomyces cerevisiae EC1118]		transmembrane transport|GO:0055085	integral component of membrane|GO:0016021
Group7	CAY82271;EGA73584;EGA81203;EGA81579;EGA84294;AEJ82629EC1118_1N26_0012p [Saccharomyces cerevisiae EC1118]	hydrolase activity|GO:0016787		
Group8	EEU07466;AEJ82628 hypothetical protein C1Q_05654 [Saccharomyces cerevisiae JAY291]	transcription factor activity|GO:0000981	regulation of transcription, DNA-templated|GO:0006355	nucleus|GO:0005634
Group9	EDV12487;CAY82274;EGA73587;EGA81575 hypothetical protein SCRG_03376 [Saccharomyces cerevisiae RM11-1a]			
Group10	EDV12565;EDZ68590;EEU04265;CAY82567;EGA86180;EHN03954 hypothetical protein SCRG_03463 [Saccharomyces cerevisiae RM11-1a]			

Group11	EDN61464;EDZ71480;EGA58282 killer toxin [Saccharomyces cerevisiae YJM789]			
Group12		transcription factor activityIGO:0000981	regulation of transcription, DNA-templatedIGO:0006355	nucleusIGO:0005634
Group14	EDV12484;CAX63268;CAY82273;EGA77666;EGA80271;EGA81576;EGA84298;AEJ82631;CDF91657 hypothetical protein SCRG_03373 [Saccharomyces cerevisiae RM11-1a]			
Group15	EDN59774 Ulp1 interacting protein [Saccharomyces cerevisiae YJM789]			
Group16	CAY81685;EGA84223;EHN03959 EC1118_1M36_0045p [Saccharomyces cerevisiae EC1118]			
Group17	CAY81684;EGA59952;EGA84224;EHN03958 EC1118_1M36_0034p [Saccharomyces cerevisiae EC1118]			
Group18	NP_040489;P03871;AAB59341;EGA80127 Rep 1 protein [Saccharomyces cerevisiae A364A]		plasmid partitioningIGO:0030541	
Group19	NP_040490;Q06891;AAB59342;EGA80128;EHM99587;EIW06932 D protein [Saccharomyces cerevisiae A364A]			
Group21	EIW07371 hypothetical protein CENPK1137D_1958 [Saccharomyces cerevisiae CEN.PK113-7D]			
Group22	EGA83446 hypothetical protein QA23_0892 [Saccharomyces cerevisiae Lalvin QA23]			
Group23	NP_040491;WP_022618806;P03872;AAB59343;EEU05475;EGA80129;CCL20271;CCL67055;CCL74498 Rep 2 protein [Saccharomyces cerevisiae A364A]			

Group24			carbon-nitrogen ligase activity, with glutamine as amido-N-donorIGO:0016884	
Group25	3LRK_A;3LRL_A Chain A, Structure Of Alfa-Galactosidase (Mel1) From Saccharomyces Cerevisiae	alpha-galactosidase activityIGO:0004557	carbohydrate metabolic processIGO:0005975	
Group29	CBK52122 hypothetical protein [Saccharomyces bayanus]	transcription factor activityIGO:0000981	regulation of transcription, DNA-templatedIGO:0006355	nucleusIGO:0005634
Group30	P40113;AAA81533;CBK52124;CBZ39537;CCA61348 RecName: Full=Protein RTM1		response to stressIGO:0006950	integral component of membraneIGO:0016021
Group31	EEU06104 hypothetical protein C1Q_05662 [Saccharomyces cerevisiae JAY291]	methyltransferase activityIGO:0008168	metabolic processIGO:0008152	
Group32		alpha-galactosidase activityIGO:0004557	carbohydrate metabolic processIGO:0005975	
Group33	BAF57236 tyrosine permease [Saccharomyces pastorianus]	amino acid transmembrane transporter activityIGO:0015171	amino acid transmembrane transportIGO:0003333	membraneIGO:0016020
Group34	EEU04262 hypothetical protein C1Q_05665 [Saccharomyces cerevisiae JAY291]		transmembrane transportIGO:0055085	integral component of membraneIGO:0016021
Group35	EDN60891 hypothetical protein SCY_5476 [Saccharomyces cerevisiae YJM789]			
Group38			transmembrane transportIGO:0055085	integral component of membraneIGO:0016021
Group39	EEU04263 hypothetical protein C1Q_05666 [Saccharomyces cerevisiae JAY291]	transcription factor activityIGO:0000981	regulation of transcription, DNA-templatedIGO:0006355	nucleusIGO:0005634
Group40	GAA26167 K7_06167p [Saccharomyces cerevisiae Kyokai no. 7]			membraneIGO:0016020
Group41	BAF95823 amidase homolog [Saccharomyces pastorianus]	carbon-nitrogen ligase activity, with glutamine as		

		amido-N-donorIGO:0016884		
Group42	EDV10953 aspartic proteinase yapsin-6 precursor [Saccharomyces cerevisiae RM11-1a]	aspartic-type endopeptidase activityIGO:0004190	proteolysisIGO:0006508	
Group43	EHN03960 hypothetical protein VIN7_5252 [Saccharomyces cerevisiae x Saccharomyces kudriavzevii VIN7]			
Group47	GAA23139 K7_02354p [Saccharomyces cerevisiae Kyokai no. 7]	N-acetyltransferase activityIGO:0008080		
Group48	GAA21449 K7_Ehl2p [Saccharomyces cerevisiae Kyokai no. 7]	cis-stilbene-oxide hydrolase activityIGO:0033961		
Group50	CAY86672;EHN03998 EC1118_1O4_6502p [Saccharomyces cerevisiae EC1118]			
Group51	CAY86671;EHN04000 EC1118_1O4_6491p [Saccharomyces cerevisiae EC1118]		transmembrane transportIGO:0055085	integral component of membraneIGO:0016021
Group52	CAY86674;EHN04001 Sod2p [Saccharomyces cerevisiae EC1118]	superoxide dismutase activityIGO:0004784;metal ion bindingIGO:0046872	oxidation-reduction processIGO:0055114;super oxide metabolic processIGO:0006801	
Group53	EHN04002 Fre7p [Saccharomyces cerevisiae x Saccharomyces kudriavzevii VIN7]	oxidoreductase activityIGO:0016491	oxidation-reduction processIGO:0055114	
Group54	CAY86676;EHN03989 EC1118_1O4_6546p [Saccharomyces cerevisiae EC1118]		oxidation-reduction processIGO:0055114;metab olic processIGO:0008152	
Group55	CAY86677 Hxt13p [Saccharomyces cerevisiae EC1118]	substrate-specific transmembrane transporter activityIGO:0022891	transmembrane transportIGO:0055085	membraneIGO:0016020
Group56	EGA80304;EHN03992 hypothetical protein QA23_5139 [Saccharomyces cerevisiae Lalvin QA23]			
Group57	CAY86679;EGA80303;EHN03994 EC1118_1O4_6579p [Saccharomyces	isomerase activityIGO:0016853	carbohydrate metabolic processIGO:0005975	

	cerevisiae EC1118]			
Group58	EHN03995 Sor1p [Saccharomyces cerevisiae x Saccharomyces kudriavzevii VIN7]	oxidoreductase activity|GO:0016491	oxidation-reduction process|GO:0055114	
Group60	EGA80321 hypothetical protein QA23_5136 [Saccharomyces cerevisiae Lalvin QA23]			
Group61	CAY86680;EGA80320 EC1118_1O4_6612p [Saccharomyces cerevisiae EC1118]	oxidoreductase activity|GO:0016491		
Group62	CAY86682;EHN03988 EC1118_1O4_6634p [Saccharomyces cerevisiae EC1118]	transmembrane transporter activity|GO:0022857	transmembrane transport|GO:0055085	integral component of membrane|GO:0016021
Group63	EDZ68613;EHN03981 hypothetical protein AWRI1631_10970060 [Saccharomyces cerevisiae AWRI1631]			
Group64	CAY86685;EGA80311;EHN03986 EC1118_1O4_6667p [Saccharomyces cerevisiae EC1118]			
Group67	EDN59775 conserved protein [Saccharomyces cerevisiae YJM789]			
Group68	EDN59776 pheromone-regulated membrane protein [Saccharomyces cerevisiae YJM789]	hydrolase activity|GO:0016787		
Group69				
Group70	GAA23025 K7_02212p [Saccharomyces cerevisiae Kyokai no. 7]			
Group71				
Group72	EHN03996 Nft1p, partial [Saccharomyces cerevisiae x Saccharomyces kudriavzevii VIN7]	ATPase activity, coupled to transmembrane movement of substances|GO:0042626	transmembrane transport|GO:0055085	integral component of membrane|GO:0016021
Group73	EHN03983 hypothetical protein VIN7_5225 [Saccharomyces cerevisiae x Saccharomyces kudriavzevii VIN7]			

Group74	EHN03987 hypothetical protein VIN7_5229 [Saccharomyces cerevisiae x Saccharomyces kudriavzevii VIN7]			
Group75		phosphotransferase activity, alcohol group as acceptor|GO:0016773;ATP binding|GO:0005524		
Group76	GAA24511 K7_11205p [Saccharomyces cerevisiae Kyokai no. 7]			
Group77		nucleic acid binding|GO:0003676		
Group80	CAY86684 EC1118_1O4_6656p [Saccharomyces cerevisiae EC1118]			
