# Supplementary material for: AGAPE (Automated Genome Analysis PipelinE) for Pan-Genome Analysis of Saccharomyces cerevisiae
Source: PLoS One. 2015 Mar 17;10(3):e0120671. doi: 10.1371/journal.pone.0120671 (PMC4363492; doi:10.1371/journal.pone.0120671)
Supplement: S3 Table — Different cutoff values were applied to construct the non-reference based trees in Fig. 5(A). The tree for each combination of the cutoff values was compared to the genome-wide SNP-based tree in Fig. 5(B) using Ktreedist. Lower K scores from Ktreedist indicate that two trees are more similar in terms of differences of the relative branch length and topology. BLAST E-value cutoff did not affect the tree topology. Similarity higher than 75% and length cutoff of 75% showed the lowest K score, so we chose 75% for both similarity and length cutoff values. (PDF) [file pone.0120671.s003.pdf]

S3 Table. Choosing appropriate cutoff values for constructing the phylogentetic tree based on presence or absence of novel genes. Different cutoff values were applied to construct the non-reference based trees in Figure 5(A). The tree for each combination of the cutoff values was compared to the genome-wide SNP-based tree in Figure 5(B) using Ktreedist (Soria-Carrasco et *al*. 2007). Lower K scores from Ktreedist indicate that two trees are more similar in terms of differences of the relative branch length and topology. BLAST E-value cutoff did not affect the tree topology. Similarity higher than 75% and length cutoff of 75% showed the lowest K score, so we chose 75% for both similarity and length cutoff values.

Similarity cutoff	Length cutoff	E-value cutoff	K score
70%	65%	1.00E-01	0.56355
70%	65%	1.00E-03	0.56355
70%	65%	1.00E-05	0.56355
70%	65%	1.00E-07	0.56355
70%	70%	1.00E-01	0.56397
70%	70%	1.00E-03	0.56397
70%	70%	1.00E-05	0.56397
70%	70%	1.00E-07	0.56397
70%	75%	1.00E-01	0.56133
70%	75%	1.00E-03	0.56133
70%	75%	1.00E-05	0.56133
70%	75%	1.00E-07	0.56133
70%	80%	1.00E-01	0.56344
70%	80%	1.00E-03	0.56344
70%	80%	1.00E-05	0.56344
70%	80%	1.00E-07	0.56344
75%	65%	1.00E-01	0.55006
75%	65%	1.00E-03	0.55006

75%	65%	1.00E-05	0.55006
75%	65%	1.00E-07	0.55006
75%	70%	1.00E-01	0.55386
75%	70%	1.00E-03	0.55386
75%	70%	1.00E-05	0.55386
75%	70%	1.00E-07	0.55386
**75%**	**75%**	**1.00E-01**	**0.54803**
**75%**	**75%**	**1.00E-03**	**0.54803**
**75%**	**75%**	**1.00E-05**	**0.54803**
**75%**	**75%**	**1.00E-07**	**0.54803**
75%	80%	1.00E-01	0.5523
75%	80%	1.00E-03	0.5523
75%	80%	1.00E-05	0.5523
75%	80%	1.00E-07	0.5523
80%	65%	1.00E-01	0.55603
80%	65%	1.00E-03	0.55603
80%	65%	1.00E-05	0.55603
80%	65%	1.00E-07	0.55603
80%	70%	1.00E-01	0.55669
80%	70%	1.00E-03	0.55669
80%	70%	1.00E-05	0.55669
80%	70%	1.00E-07	0.55669
80%	75%	1.00E-01	0.55373

80%	75%	1.00E-03	0.55373
80%	75%	1.00E-05	0.55373
80%	75%	1.00E-07	0.55373
80%	80%	1.00E-01	0.55713
80%	80%	1.00E-03	0.55713
80%	80%	1.00E-05	0.55713
80%	80%	1.00E-07	0.55713
85%	65%	1.00E-01	0.55729
85%	65%	1.00E-03	0.55729
85%	65%	1.00E-05	0.55729
85%	65%	1.00E-07	0.55729
85%	70%	1.00E-01	0.56093
85%	70%	1.00E-03	0.56093
85%	70%	1.00E-05	0.56093
85%	70%	1.00E-07	0.56093
85%	80%	1.00E-01	0.56086
85%	80%	1.00E-03	0.56086
85%	80%	1.00E-05	0.56086
85%	80%	1.00E-07	0.56086
85%	90%	1.00E-01	0.56086
85%	90%	1.00E-03	0.56086
85%	90%	1.00E-05	0.56086
85%	90%	1.00E-07	0.56086

References

Soria-Carrasco V, Talavera G, Igea J, Castresana J. (2007). The K tree score: quantification of differences in the relative branch length and topology of phylogenetic trees. Bioinformatics 23, 2954-2956.
