# Supplementary material for: AGAPE (Automated Genome Analysis PipelinE) for Pan-Genome Analysis of Saccharomyces cerevisiae
Source: PLoS One. 2015 Mar 17;10(3):e0120671. doi: 10.1371/journal.pone.0120671 (PMC4363492; doi:10.1371/journal.pone.0120671)
Supplement: S4 Table — We counted the number of phenotypes per strain as reported in SGD and chose to sequence those strains with the highest phenotype counts. Note that we have grouped all four of the S288C-identical strains (BY4741, BY4742, FY1679 and X2180) into one class called “S288C”. In addition to the strains listed in this table, we chose several other strains to sequence as described in the main text. (PDF) [file pone.0120671.s004.pdf]

S4 Table. Phenotype count per strain. We counted the number of phenotypes per strain as reported in SGD and chose to sequence those strains with the highest phenotype counts. Note that we have grouped all four of the S288C-identical strains (BY4741, BY4742, FY1679 and X2180) into one class called "S288C". In addition to the strains listed in this table, we chose several other strains to sequence as described in the main text.

Strain	Count	Percent
RM11-1a	2	0.002
Y55	18	0.015
FL100	57	0.046
JK9-3d	111	0.09
CEN.PK	213	0.174
X2180-1A	276	0.225
D273-10B	278	0.227
SEY6210	414	0.337
SK1	859	0.7
Sigma1278b	2170	1.768
W303	3158	2.573
Other	11123	9.063
S288C	104045	84.78
