# Supplementary material for: AGAPE (Automated Genome Analysis PipelinE) for Pan-Genome Analysis of Saccharomyces cerevisiae
Source: PLoS One. 2015 Mar 17;10(3):e0120671. doi: 10.1371/journal.pone.0120671 (PMC4363492; doi:10.1371/journal.pone.0120671)
Supplement: S1 Fig — Copy number variants (CNVs) were called by the program “CNVnator” based on read coverage depth of each strain genome relative to the reference genome. Genomic intervals identified as CNVs in each strain were visualized as blue boxes using the IGV (Integrative Genomics Viewer) tool. (PDF) [file pone.0120671.s005.pdf]

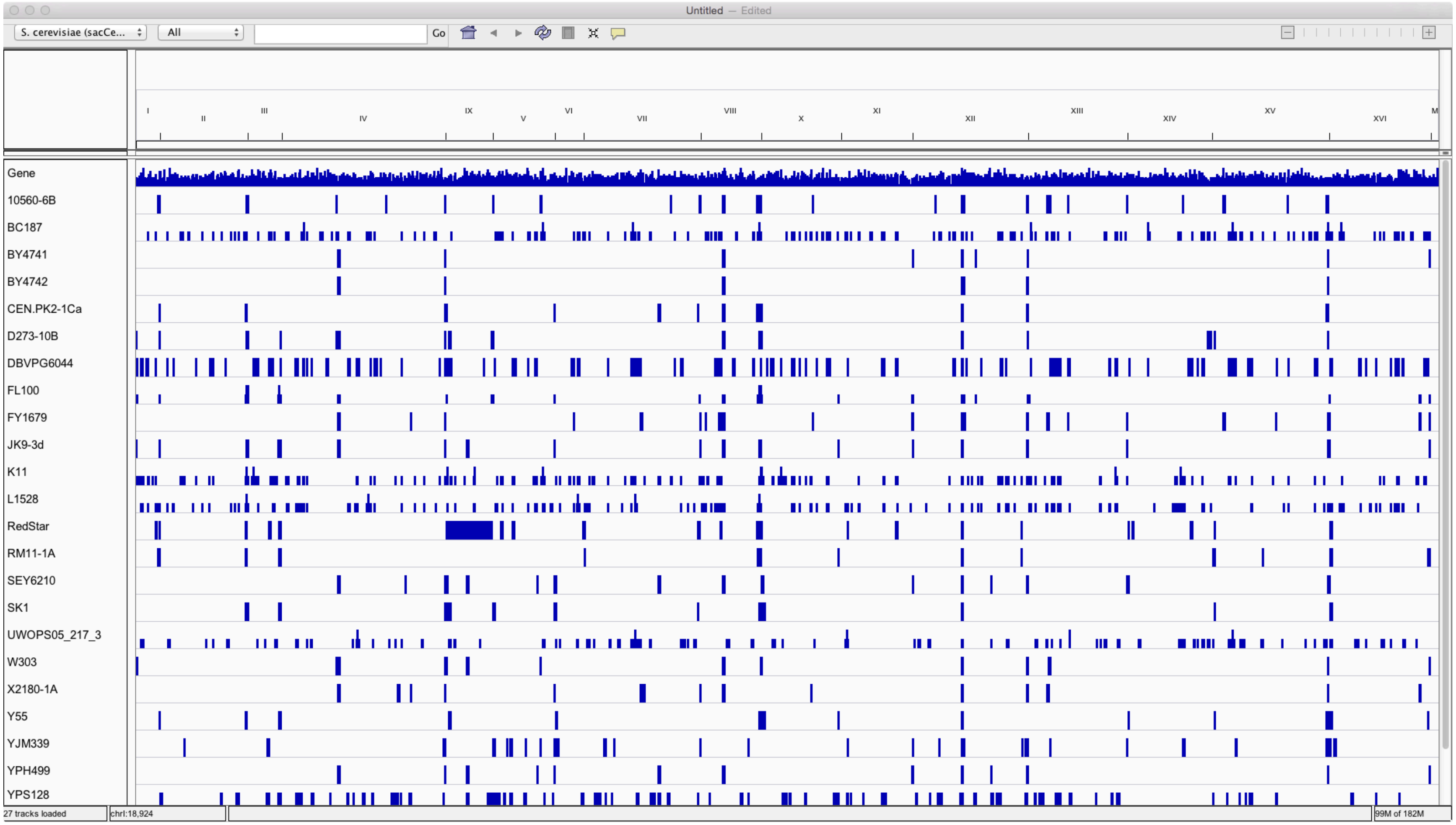

S1 Fig. Copy number variants in *S. cerevisiae*. Copy number variants (CNVs) were called by the program "CNVnator" based on read coverage depth of each strain genome relative to the reference genome (Abyzov *et al*. 2011). Genomic intervals identified as CNVs in each strain were visualized as blue boxes using the IGV (Integrative Genomics Viewer) tool (Thorvaldsdóttir *et al*. 2012).

References

Abyzov A, Urban AE, Snyder M, Gerstein M. (2011) CNVnator: an approach to discover, genotype, and characterize typical and atypical CNVs from family and population genome sequencing. Genome Res 21(6):974-84.

Thorvaldsdóttir H, Robinson J, Mesirov J**.** (2012) Integrative Genomics Viewer (IGV): high-performance genomics data visualization and exploration. Briefings in Bioinformatics 14(2).
