# Supplementary material for: AGAPE (Automated Genome Analysis PipelinE) for Pan-Genome Analysis of Saccharomyces cerevisiae
Source: PLoS One. 2015 Mar 17;10(3):e0120671. doi: 10.1371/journal.pone.0120671 (PMC4363492; doi:10.1371/journal.pone.0120671)
Supplement: S2 Fig — All the assembly contigs of each strain genome were aligned to the reference genome using LASTZ. The alignments were visualized using the IGV tool. Since all the alignments for other chromosomes are available, users can easily view assembly coverage in other chromosomes with IGV. (PDF) [file pone.0120671.s006.pdf]

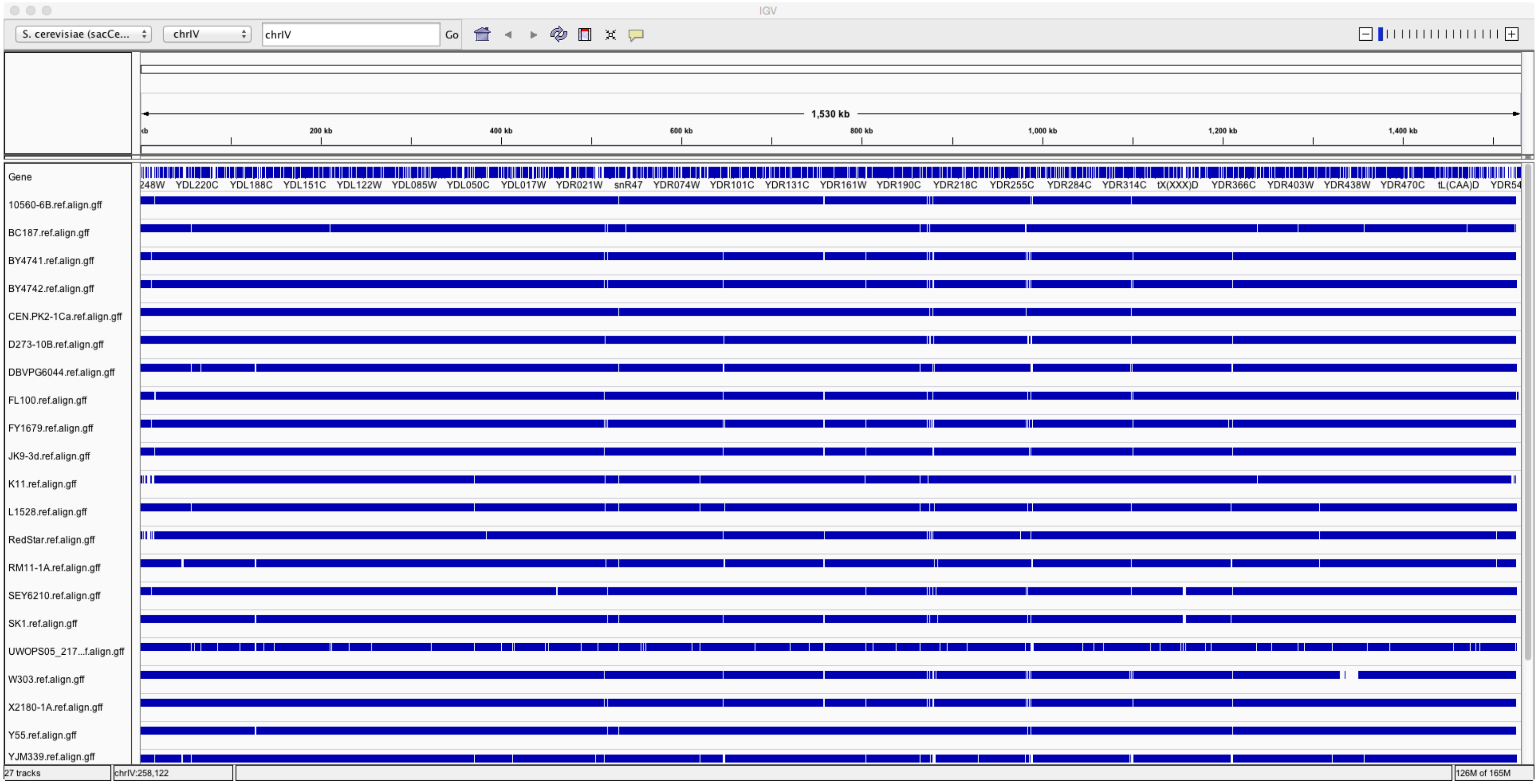

S2 Fig. Assembly coverage in chromosome IV. All the assembly contigs of each strain genome were aligned to the reference genome using LASTZ. The alignments were visualized using the IGV tool (Thorvaldsdóttir *et al*. 2012). Since all the alignments for other chromosomes are available, users can easily view assembly coverage in other chromosomes with IGV.

References

Thorvaldsdóttir H, Robinson J, Mesirov J**.** (2012) Integrative Genomics Viewer (IGV): high-performance genomics data visualization and exploration. Briefings in Bioinformatics 14(2).
